# Inducible Expression of Mu-Class Glutathione *S*-Transferases Is Associated with Fenpropathrin Resistance in *Tetranychus cinnabarinus*

**DOI:** 10.3390/ijms151222626

**Published:** 2014-12-08

**Authors:** Guang-Mao Shen, Li Shi, Zhi-Feng Xu, Lin He

**Affiliations:** Key Laboratory of Entomology and Pest Control Engineering, College of Plant Protection, Southwest University, Chongqing 400715, China; E-Mails: blackaet@163.com (G.-M.S.); shiliabc@126.com (L.S.); xuzhifeng923@126.com (Z.-F.X.)

**Keywords:** *Tetranychus cinnabarinus*, glutathione *S*-transferases (GSTs), resistance, fenpropathrin

## Abstract

The carmine spider mite, *Tetranychus cinnabarinus* (Boisduval), is a serious pest on a variety of economically important crops widely distributed in China, and its resistance to acaricides has quickly developed. In this study, we fully sequenced 13 GST genes of *T. cinnabarinus* (TcGSTs). The phylogenetic tree showed that five of them belonged to the delta class and the other eight belonged to the mu class. The alignment of gene sequences and comparison of gene expressions between a fenpropathrin-resistant strain (FR) and a susceptible strain (SS) showed that neither point mutation nor overexpression was detected in TcGSTs. However, when challenged by a sublethal dose of fenpropathrin, the mRNA levels of three GSTs from the mu class (*TCGSTM2*, *TCGSTM3*, and *TCGSTM8*) highly increased in FR, while in SS, the expression of these genes was still at the same level under the treatment. In conclusion, specific TcGSTs were identified that were inducible to stimulation by fenpropathrin, and proved that TcGSTs in FR were not constantly expressed at a high level, but could react much more quickly under the stress of fenpropathrin than SS.

## 1. Introduction

The carmine spider mite, *Tetranychus cinnabarinus*, is one of the major pest species on agriculture crops in China [1]. As a polyphagous pest, *T. cinnabarinus* mainly feeds on economically important plants, such as vegetables, cotton, tobacco, and maize, and causes significant decrease in quality and yield [2]. Since its morphological, biological, and molecular characteristics are quite similar to that of the two-spotted mite, *Tetranychus urticae*, there is some controversy about their species status [3,4]. In this situation, *T. cinnabarinus* is also considered as the red form of *T. urticae* by some researchers [5].

Currently, chemical reagents are still used as a primary method of controlling this pest. Various acaricides with different action modes are applied in the fields. Organophosphates, abamectin, and pyrethroids act on the acetylcholinesterase (AChE), the glutamate-gated chloride channel, and the sodium channel, respectively [6,7,8]. Pyridaben works as a mitochondrial electron transport inhibitor [9], and cyflumetofen, a novel class of acaricide, is a strong inhibitor of the electron-transport function of complex II of the mites [10]. However, *T. cinnabarinus* has developed high resistance to these acaricides [11]. Monitoring of acaricide resistance in China shows that field strains of *T. cinnabarinus* have become insensitive to fenpropathrin [12]. In this case, research on the resistance mechanism of fenpropathrin is necessary for implementation of effective control of this pest.

It is commonly known that mutations on the target sites of acaricides orenhanced detoxification systems of pests are involved in the resistance mechanism. In the fenpropathrin resistance study of mites, a mutation in the sodium channel gene (F1538I), which was the target of fenpropathrin, resulted in resistance to this acaricide, and it was further developed as a molecular marker for resistance monitoring [13,14]. In addition, studies on the detoxification system of *T. cinnabarinus* identified that the activity of glutathione *S*-transferases (GSTs) increased in the fenpropathrin-resistant (FR) strain when compared to the susceptible strain (SS) [15]. This shows a correlation between GSTs and fenpropathrin resistance. However, there is still insufficient information about the specific function of GSTs in *T. cinnabarinus*.

In insects, the function of GSTs has been widely studied at the molecular level [16]. As a large family of multifunctional enzymes, special attention is paid to the delta and epsilon classes of insect GSTs. These two classes are distinctive in insects, and most members of them are proved to be capable of metabolizing insecticides. Studies have demonstrated that the increased expression of *LmGSTd1* contributed to the resistance of locust *Locusta migratoria* to organophosphate pesticides [17], and under the exposure of β-cypermethrin, the mRNA levels of GSTs from epsilon class were up-regulated in *Bactrocera dorsalis* [18]*.* In *T. cinnabarinus*, although either synergist or enzyme activity tests showed a correlation between TcGSTs and resistance, the relation of specific TcGSTs to its resistance has not been clarified yet [14]. The genome annotation of *T. urticae* and transcriptome analysis of *T. cinnabarinus* provides an opportunity to clarify the specific role of TcGSTs in the resistance mechanisms [19,20].

In this study, we fully sequenced 13 TcGSTs; Five of them were placed in the delta class and the other eight in the mu class. The qPCR results showed that these TcGSTs have specific expression patterns in different developmental stages. The alignment of gene sequences between FR and SS showed no difference. The mRNA levels of TcGSTs, either from the delta or muclass, were slightly increased in the fenpropathrin-resistant strain (FR) over the susceptible strain (SS), but did not reach a significant level. However, when exposed to fenpropathrin at LC_30_ (sub-lethal concentration), the mRNA levels of three TcGSTs from the mu class (*TCGSTM2*, *TCGSTM3*, and *TCGSTM8*) highly increased in FR, while their expressions did not change in SS under the same treatment. The results showed that the TcGSTs did not constantly overexpress in the resistant strain, but special TcGSTs were sensitive to the stimulation of fenpropathrin and could express at a high level timely to prevent damage.

## 2. Results and Discussion

### 2.1. Bioassay Results

The bioassay results of fenpropathrin showed that the LC_50_ of FR was 61,581.93 mg/L. Comparing with the LC_50_ (median lethal concentration) of SS (606.98 mg/L), the resistance ratio (RR, FR/SS) was about 101-fold. In addition, the LC_30_ of FR and SS was calculated (280.63 mg/L for SS, and 27,710.96 mg/L for FR), and used as a sublethal dose to treat the mites (Table 1). The LC_50_ of both SS and FR presented here was higher than some bio-assay results of fenpropathrin on mites, which might be due to the difference of bioassay methods [10]. The LC_50_ of both FR and SS was in accordance with our previous study, and the F1538I substitution only found in FR, which indicated that SS remains susceptible to fenpopathrin [7].

**Table 1:** The susceptibility of fenpropathrin-resistant strain (FR) and susceptible strain (SS) to fenpropathrin.

Strains	LC_50_ (mg/L) 95% CI	LC_30_ (mg/L) 95% CI	Slope (±SE)	RR
SS	606.98 (490.80–727.67)	280.63 (188.04–364.23)	1.57 ± 0.21	–
FR	61,581.93 (49,641.61–75,068.16)	27,710.96 (18,492.14–36,037.43)	1.51 ± 0.21	101

LC_50_: 50% lethal concentration; LC_30_: 30% lethal concentration; SE: Standard error; RR: Resistance ratio.

### 2.2. Sequencing and Annotation of GSTs from T. cinnabarinus

**Figure 1:**
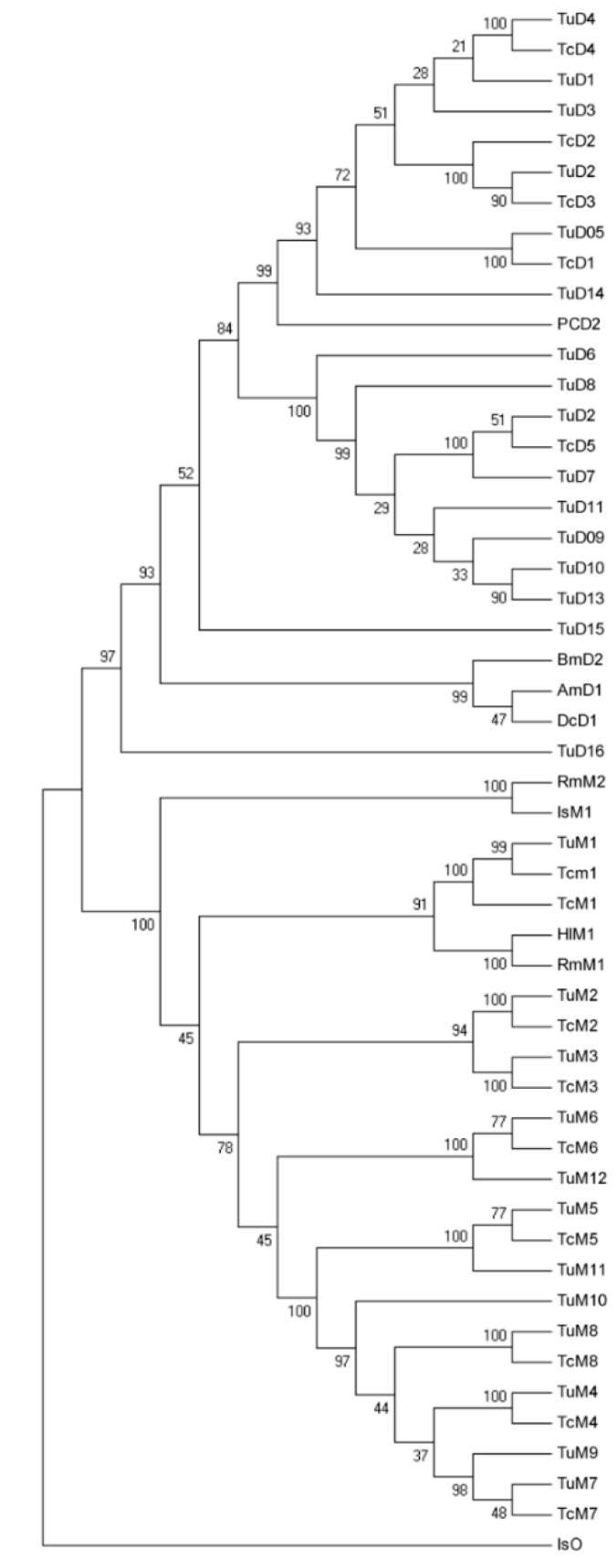
Neighbor-joining phylogenetic analysis of TcGSTs. Reference sequences were obtained from the NCBI database and the genome database of *Tetranychus urticae*. Pc: *Panonychus citri* (AFD36890, AFD36888). Tu: *Tetranychus urticae* (tetur01g02230, tetur01g02470, tetur01g02480, tetur01g02500, tetur01g02510, tetur03g07920, tetur26g01460, tetur26g02801, tetur26g02802, tetur26g01490, tetur26g01500, tetur26g01510, tetur29g00220, tetur31g01330, tetur31g01390, tetur03g09230, tetur05g05180, tetur05g05190, tetur05g05200, tetur05g05210, tetur05g05220, tetur05g05240, tetur05g05250, tetur05g05260, tetur05g05270, tetur05g05290, tetur05g05300). Hl: *Haemaphysalis longicornis* (AAQ74441). Bm: *Bombyx mori* (NP_001036974). Am: *Apis mellifera* (NP_001171499). Dc: *Drosophila conformis* (AGY31901). Rm: *Rhipicephalus microplus* (AAD15991, AHH29555, AAL99403). Is: *Ixodes scapularis* (EEC09568, EEC09568, EEC09120).

According to the transcriptome data of *T. cinnabarinus* and the genome of *T. urticae*, the full length of 13 TcGSTs containing complete open reading frames of *T. cinnabarinus* were cloned and sequenced. The length of nucleotide sequences ranged from 648 to 699 bp, and the molecular weight of the predicted proteins ranged from 24.169 to 26.634 kD. The sequences were further aligned with the nucleotide sequences in NCBI and the genome database of *T. urticae*. These 13 TcGSTs were classified into two classes according to the specific hits of putative conserved domains in the NR database (http://www.ncbi.nlm.nih.gov/). Five of them contained the specific domain of the “delta” class, and were named “*TcGSTD1–5*”. The other eight were classified into “mu” class, and named “*TcGSTM1–8*”. Sequence information was submitted to GenBank and the accession numbers for each gene were as follows: KM076629–KM076633 for *TcGSTD1*–*5*, and KM076634–KM076641 for *TcGSTM1–8*. The similarity of deduced amino acid sequences of identified delta class GSTs was relatively high, especially for *TcGSTD2* and *TcGSTD3* (85.65%) (Table 2), and the similarity of mu class GSTs was 37.50%–73.57% (Table 3). A phylogenetic tree was constructed with GST sequences from Acarina (*Rhipicephalus microplus*, *Ixodes scapularis*, *Haemaphysalis longicornis*, *Panonychus citri*, and *T. urticae*) and insects (*Bombyx mori*, *Apis* *mellifera*, and *Drosophila conformis*). To clarify the relation between TcGSTs and TuGSTs, all GST sequences of delta and mu classes from *T. urticae* were included (Figure 1). As results, this work successfully fully sequenced five delta-class GSTs and eight mu-class GSTs. Compared with the genome of *T. urticae*, in which 32 GSTs were classified, including 16 delta-class GSTs and 12 mu-class GSTs [19]. Identification of other TcGSTs was still necessary to get a comprehensive view of these two classes. From the qPCR results, we think low expression of TcGSTs might be the major obstacle for amplification.

**Table 2:** Sequence similarity among TcGSTs from the delta class.

Gene	D2	D3	D4	D5
D1	76.85%	75.93%	79.63%	46.91%
D2	–	85.65%	74.07%	45.66%
D3	–	–	71.76%	43.83%
D4	–	–	–	44.50%

**Table 3:** Sequence similarity among TcGSTs from the mu class.

Gene	M2	M3	M4	M5	M6	M7	M8
M1	37.5%	38.63%	39.65%	37.89%	40.17%	39.47%	41.41%
M2	–	66.38%	46.61%	40.44%	45.78%	40.09%	39.65%
M3	–	–	40.68%	39.41%	41.18%	41.53%	38.98%
M4	–	–	–	59.03%	47.60%	73.57%	69.60%
M5	–	–	–	–	40.03%	63.00%	60.79%
M6	–	–	–	–	–	44.10%	41.92%
M7	–	–	–	–	–	–	72.96%

As an important enzyme, GSTs were found in most plants, mammals, insects, ticks, and mites. Detoxification of both endogenous and xenobiotic toxins was the primary function of GSTs [20]. GSTs were dimeric proteins, which consisted of two domains, with a *N*-terminal thioredoxin (TRX)-fold domain and a *C*-terminal alpha helical domain. The glutathione (GSH) binding site (G-site), a common active site of GST, was located in the *N*-terminal TRX-fold domain, and a xenobiotic binding site (H-site), which varied between different classes and isotypes, was located in the *C*-terminal alpha helical domain. The sequence feature of these two domains was important to separate single GST genes into different classes [21]. In insects, majority of GSTs belong to six classes, including delta, epsilon, zeta, omega, theta, and sigma. Delta and epsilon were unique to insects and also the biggest two classes. GSTs from the delta and epsilon classes could facilitate reductive dehydrochlorination of insecticides by conjugating them with GSH to produce water-soluble metabolites that could be easily excreted [16]. The classification of GSTs in mites was quite different from that in insects. In *T. urticae*, 32 GSTs were classified into five classes, including delta, mu, omega, zeta, and kappa. The delta class was the biggest group, followed by the μ class, which was absent in insects [19]. It was interesting that in Acari, *Ixodes scapularis* had all three special GST classes, delta, epsilon, and mu, but in the mites, such as *T. urticae* or *P. citri*, no epsilon GSTs were identified; instead, the mu class was an important part [22]. The transcriptome data for *T. cinnabarinus* also indicate that the delta and mu classes were the biggest two groups, and no GSTs were classified as epsilon [23]. In fact, mu-class GSTs were widely studied in vertebrates. They were highly enriched in the human liver, skeletal muscle, and brain. Their functions were related to disease resistance, including cancer and neurodegenerative disorders [24]. In contrast, although mu-class GSTs were considered as a key point in acaricide resistance, studies about their specific molecular characterization were still insufficient. As a special detoxification enzyme in mites, it was meaningful to clarify the function of TcGSTs and enrich the resistance mechanism study of *T. cinnabarinus* from the aspect of detoxification.

Meanwhile, at least 10 single clones for each of 13 identified TcGSTs were sequenced from SS and FR, and the alignment results showed that no constant amino acid substitution was found between SS and FR.

### 2.3. Specific Expression of TcGSTs in Different Developmental Stages

**Figure 2:**
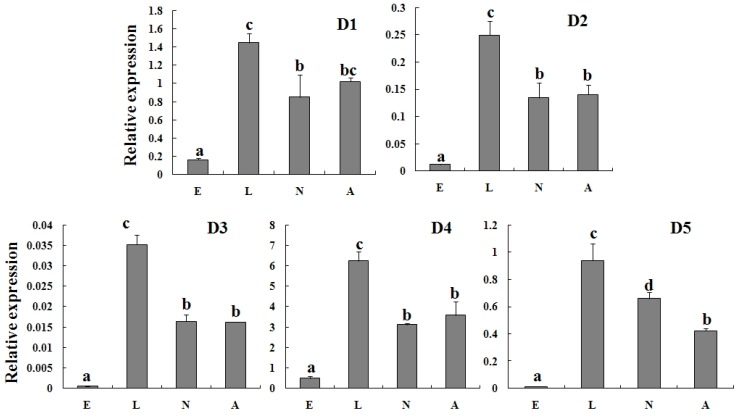
Relative expression of five delta-class (D1–D5) TcGSTs in different developmental stages. Different letters above each bar indicate statistical difference determined by ANOVA analysis and the Duncan’s Multiple Range Test (*p* < 0.05). E: Eggs; L: Larvae; N: Nymphs; A: Adults.

**Figure 3:**
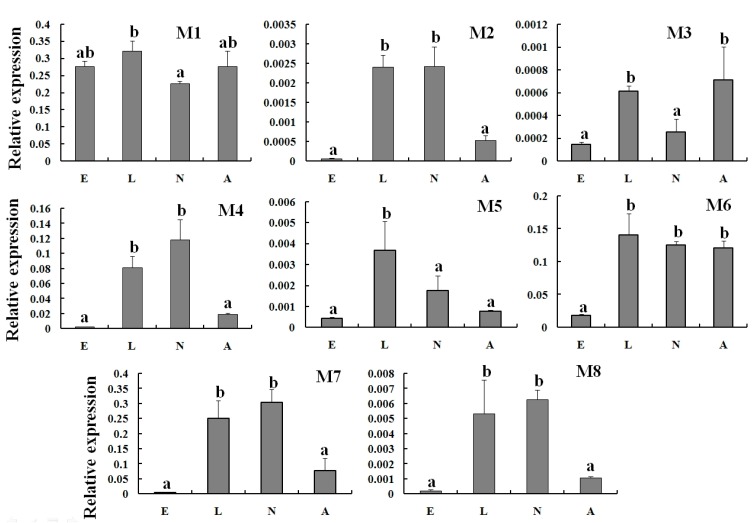
Relative expression of eight mu-class (M1–M8) TcGSTs in different developmental stages. Different letters above each bar indicate statistical difference determined by ANOVA analysis and the Duncan’s Multiple Range Test (*p* < 0.05). E: Eggs; L: Larvae; N: Nymphs; A: Adults.

To clarify the expression characterization of TcGSTs, RT-qPCR was used to detect the transcripts of each TcGST gene among different developmental stages (eggs, larvae, nymphs, and adults). The results showed that the expression profiles of TcGSTs were quite specific in different developmental stages. The TcGSTs from the delta class were abundant in larvae, about 1.42- to 2.22-fold higher than that in nymphs or adults, and lowest in eggs. The mRNA levels in nymphs and adults were basically the same. The expression profiles of five delta GST genes were in accordance with this tendency (Figure 2). Comparatively, the mRNA levels of TcGSTs from the mu class in developmental stages were different (Figure 3). The most special one was *TcGSTM1.* It was highly expressed in all life stages, including eggs; on the contrary, the expression profiles of other GSTs were quite low in eggs. The expression of *TcGSTM3* was high in larvae and adults, but relatively low in eggs and nymphs. *TcGSTM6* was abundant in larvae, nymphs, and adults, and only insufficient in eggs. The expression of *TcGSTM5* was only high in larvae, but in eggs, nymphs, and adults was at a relatively low level. The other four mu class GSTs (*TcGSTM2*, *TcGSTM4*, *TcGSTM7*, and *TcGSTM8*) were all highly expressed in larvae and nymphs, but were relatively low in eggs and adults.

The expression of GST genes was not only altered by exogenous toxin, but also by endogenous factors. Hormones or cytokines could regulate the expression of GSTs under physiological and pathophysiological conditions. A study of *B. mori* showed that the expression of GSTs in larvae midgut was regulated by JHA (juvenile hormone analogue) and 20E (20-Hydroxyecdysone) treatment [25]. The mRNA level of GSTs might be related to the JHA and 20E concentrations in developmental stages. In fact, the expression of GSTs during developmental stages was quite specific in different species. The expressions of most GSTs from *L. migratoria* did not change much among developmental stages, while some of them expressed at a low level in eggs [26]. In *P. citri*, seven GST genes expressed at a relatively high level in eggs, except for *PcGSTm1* [23]. But in *T. cinnabarinus*, TcGSTs either belong to the delta class or the mu class, all highly expressed in larval and nymph stages. These two stages were accompanied by dramatic changes in phenotype. The results suggested that the TcGSTs from the delta class were important for larvae, and the other mu class TcGSTs may play different roles in each developmental stage. The relation between the expression of GSTs and the development of organisms was an interesting point. However, currently only a few works have focused on this aspect. Our work has provided some basic information for further study to reveal the function of GSTs in development.

### 2.4. Expression Profiles of TcGSTs between Fenpropathrin-Resistant (FR) and Susceptible Strains (SS)

**Figure 4:**
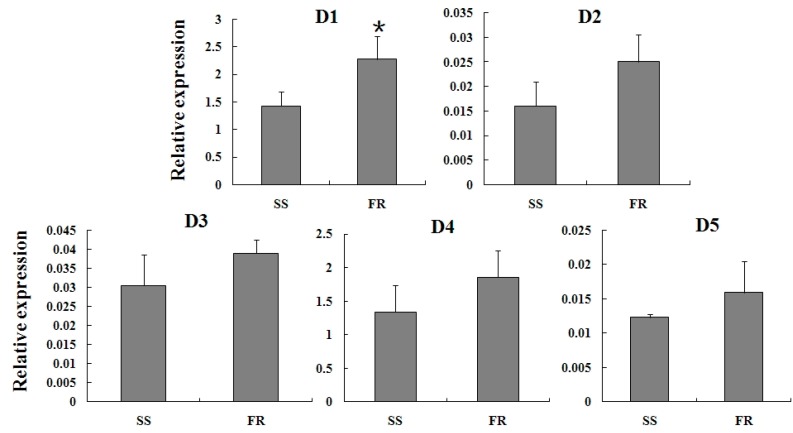
Relative expression of five delta-class (D1–D5) TcGSTs in female adults of SS and FR. Star above each bar indicate statistical difference determined by *t* Test (*p* < 0.05). SS: Susceptible strain; and FR: Fenpropathrin-resistant strain.

**Figure 5:**
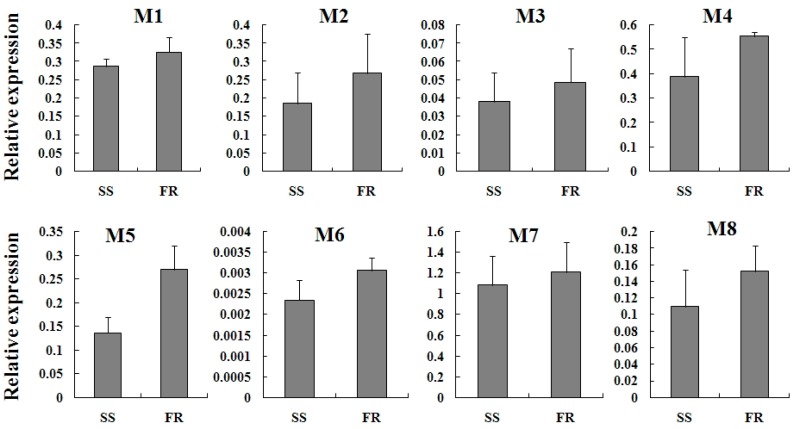
Relative expression of eight mu-class (M1–M8) TcGSTs in female adults of SS and FR. SS: Susceptible strain; and FR: Fenpropathrin-resistant strain.

The mRNA levels of TcGSTs were detected between fenpropathrin-resistant (FR) and susceptible strains (SS). The results showed that the expression of all delta-class TcGSTs was slightly increased, but did not reach a significant level except for *TcGSTD1* (Figure 4)*.* The same situation was found for the expression of mu-class TcGSTs as well (Figure 5).

Overexpression of detoxification enzymes was a major factor in resistance mechanisms [27]. The mRNA levels of P450s, GSTs, and CarE were commonly high in the resistant strains. It was beneficial for the organisms to prevent damage from insecticides effectively. In a fenvalerate-resistant strain of *Helicoverpa armigera*, the expression level of esterase gene *CCE001a* was found significantly increased in fat body, and it was considered to be associated with resistance [28]. In mosquitoes, multiple P450 genes were found to be involved in permethrin resistance, but their expression was not only up-regulated, but sometimes down-regulated in resistant strains [29]. The expression changes could be co-responsible for detoxification of insecticides. In a study of the relation between GSTs and pyrethroid insecticides, two epsilon genes were identified in *Aedes aegypti*, and the expression detection and RNAi results showed that these two genes were involved in conferring resistance to the pyrethroid [30]. Compared with CarEs and P450s, the role of GSTs in pyrethroid resistance has not been well clarified, especially at the molecular level. The synergistic effect on the FR has revealed that GSTs were important in conferring fenpropathrin resistance to *T. cinnabarinus*, and the activity of GSTs was increased in FR [14]. This was in accordance with the gene expression results. However, as the expression of all 13 *TcGSTs* was only slightly up-regulated, we wonder whether distinctive TCGSTs exist that are much more sensitive to the stimulation of fenpropathrin.

### 2.5. Expression Reaction of TCGSTs to Fenpropathrin Stimulation

**Figure 6:**
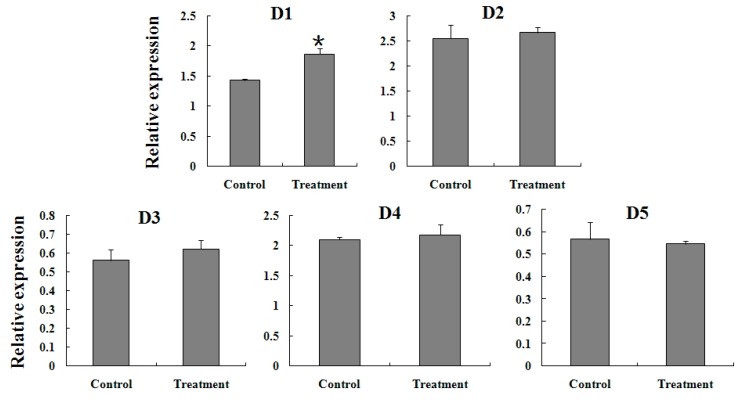
Expression reaction of five delta-class (D1–D5) TcGSTs in female adults of FR to fenpropathrin stimulation. Star above each bar indicate statistical difference determined by *t* Test (*p* < 0.05).

**Figure 7:**
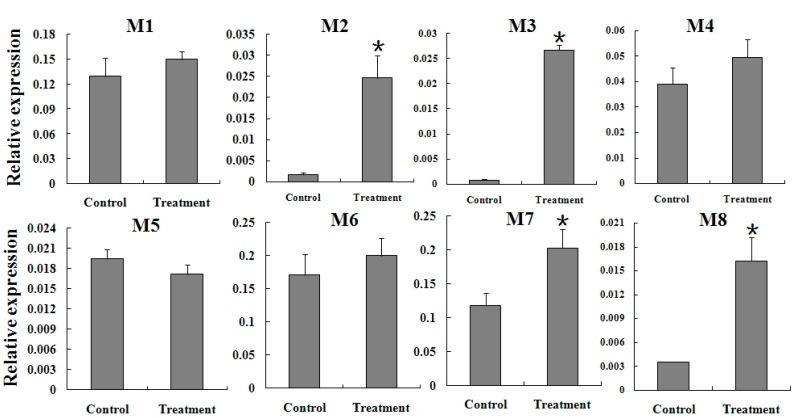
Expression reaction of eight mu-class (M1–M8) TcGSTs in female adults of FR to fenpropathrin stimulation. Star above each bar indicate statistical difference determined by *t* Test (*p* < 0.05).

To further clarify the correlation between TCGSTs and fenpropathrin resistance, a sublethal dose (LC_30_) was used to stimulate the mites from FR. After 6 h, the expression of *TCGSTM2*, *TCGSTM3*, *TCGSTM7* and *TCGSTM8* were significantly increased; In addition, the increase of *TcGSTD1* also reached a significant level. The expression profiles of other TcGSTs showed no apparent difference after fenpropathrin stimulation (Figure 6 and Figure 7) .

**Figure 8:**
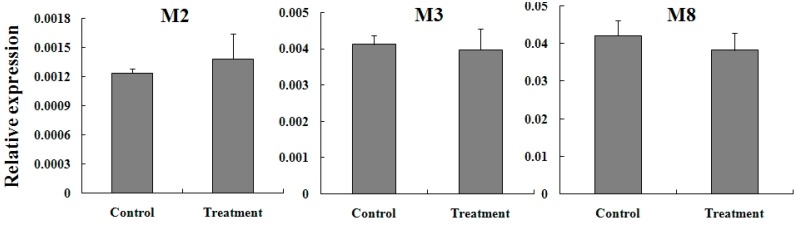
Expression reaction of *TcGSTM2*, *TcGSTM3*, and *TcGSTM8* in female adults of SS to fenpropathrin stimulation.

To confirm whether the increased expression of these GST genes was specific to FR, the expression change of three mu-class TcGSTs (*TCGSTM2*, *TCGSTM3* and *TCGSTM8*) was detected in SS after treatment by LC_30_. The mRNA levels of all three genes were not affected by treatment with fenpropathrin in SS after 6 h (Figure 8).

Although mRNA levels of all 13 TcGSTs were slightly higher in FR, they did not show a significant difference, which means overexpression of TcGSTs may not be a main factor in resistance to fenpropathrin. From the results of sublethal dose treatment, we found that expression of five TcGSTs was inducible in FR, especially three mu-class TCGSTs (*TCGSTM2*, *TCGSTM3*, and *TCGSTM8*). Their expression reached a relatively high level in a short time after stimulation by fenpropathrin. The TcGSTs from the mu class may play a more important role in detoxification of fenpropathrin. It was interesting that the sequence similarity between the inducible TcGSTs was high, 72.96% for *TCGSTM7* and *TCGSTM8*, and 66.38% for *TCGSTM2* and *TCGSTM3*. They could have common catalytic properties, and more work will be done to clarify their catalytic functions inacaricides. Identification of these genes provided useful information for further study of the detoxification system of *T. cinnabarinus*.

Meanwhile, in SS, we found these three genes were not inducible under the sublethal dose. This difference suggested that it was an important mechanism for the FR to survive from fenpropathrin, and that the expression of specific detoxification genes is much more sensitive to the stimulation of acaricides, which showed a strong reaction in a short time to prevent damage. This seems a remarkable strategy for the population growth of FR. It gives timely help when their survival is threatened, but under normal circumstance, these genes would stay stable. Constitutive overexpression of genes encoding detoxification enzymes was an important mechanism for resistance, but such action consumed extra energy even when there was no insecticide in the environment. It would affect reproduction, impair dispersal ability, and have several other effects on the insect’s fitness [31]. Different from constant overexpression, expressing detoxification genes in a timely manner was a more efficient way of utilizing energy.

Furthermore, the expression model change may come from the mutations in the promoter site or *cis*-regulatory elements (CRE). In *Drosophila melanogaster*, a distinct CRE was identified and proved to be required for the induction response of *Cyp6g1* to the xenobiotic Phenobarbital (PB) [32]. Since the CRE of GSTs has not yet been classified, special attention will be paid to the differences in the 5'-promoter or 3'-untranslated region (UTR) between FR and SS.

It is also noticed that the typical resistance mechanism not only involves increases in the metabolic capabilities of detoxification enzymes, but also decreases in target site sensitivity [27]. In our previous study on the same fenpropathrin-resistant strain, a mutation (F1538I) on the sodium channel gene, which is the target gene of fenpropathrin, played an important role in fenpropathrin resistance [7]. The mutation may generally lead to insensitivity to the acaricide without disrupting the normal function of the target. On the other hand, the metabolic detoxification system works as a supporting or major resistance component as well. Compared to the insensitivity of the target site, the function of the detoxification system is to metabolize the acaricide. We believe that the interaction and individual contribution of these two systems is a good point for further study.

## 3. Experimental Section

### 3.1. Mites

The original strain of *T. cinnabarinus* was collected in the field of Beibei District, Chongqing, China, and was reared on cowpea leaves over 13 years in an artificial climate chamber. The environmental condition was set as: 26 ± 1 °C temperature, 35%–55% humidity, and 14:10 (L:D) photoperiod. The mites kept away from any acaricides were considered to be the susceptible strain (SS). The fenpropathrin-resistant strain (FR) was continuously selected from the SS under a pressure of 70% mortality for more than 38 generations. Mites of different developmental stages were collected using the method of Xu *et al*. [24], enough eggs, larvae, nymphs, and female adults from FR were prepared for RNA extraction. Each stage contained three replications.

### 3.2. Bioassay and Acaricide Stimulation

The sensitivity of *T. cinnabarinus* was tested using the modified residual coated vial (RCV) method described by Feng *et al*. [2], briefly, fenpropathrin was dissolved in acetone to at least seven concentrations (mortality between 20% and 80%), then the solutions were transferred into 2 mL centrifuge tubes and mixed well. Redundant solutions were discarded, and tubes were placed in a fume hood until the residuals were completely evaporated. Thirty 3- to 5-day-old adult females were transferred into a fenpropathrin-coated tube. The mites were checked under an anatomical microscope after 24 h treatment. Mites were considered dead if the legs did not move when touched by a tiny brush. Each concentration was repeated three times. The lethal concentrations were calculated by Polo Plus.

To test the reaction of mites to fenpropathrin stimulation, *T. cinnabarinus* from SS and FR were treated for 6 h using LC_30_ as described above; a treatment without fenpropathrin was used as a control. The survivors were collected for RNA extraction. Each treatment contained three replications.

### 3.3. RNA Extraction and cDNA Synthesis

Different samples (mites from different strains, developmental stages, acaricide stimulation) were homogenized immediately after collection using liquid nitrogen in a mortar. RNA was extracted using TRIZOL^®^ Reagent (Life Technologies, Frederick, MD, USA) following the manufacturer’s instructions.

RNA was quantified by measuring the absorbance at 260 nm using a NanoVue UV-Vis spectrophotometer (GE Healthcare Bio-Science, Uppsala, Sweden). The purity of all RNA samples was assessed at an absorbance ratio of OD_260/280_ and at OD_260/230_, and the integrity of RNA was checked with 1% agarose gel electrophoresis. To exclude the interference of genomic DNA in qPCR, DNase I (Promega, Madison, WI, USA) was used to remove the genomic DNA in RNA.

First strand cDNA was synthesized from 1 μg of DNA-free RNA using a PrimeScript^®^ RT reagent kit (Takara, Shiga, Japan). Briefly, the 20 μL reaction system consisted of 1 μg RNA, 400 pmol Random 6 mers, 4 μL reverse transcription buffer, 2 μL PrimerScript^®^ RT Enzyme Mix I, and RNase-free H_2_O. The reverse transcription reaction was performed on a C1000TM Thermal Cycler (Bio-Rad, Foster City, CA, USA). The reaction conditions included steps at 37 °C for 15 min and 85 °C for 5 s. After the reverse transcription, synthesized cDNA was stored at −20 °C for future use.

### 3.4. Cloning of Full-Length GST cDNA and Mutation Alignment

Based on the transcriptome data of *T. cinnabarinus* and the genome database of *T. urticae* (http://bioinformatics.psb.ugent.be/orcae/overview/Tetur), sequence information of GSTs were screened out, and gene-specific primers were designed and synthesized to directly amplify the complete open reading frame for each gene. Primer information is presented in Table S1. The PCR conditions were set as follows: 3 min at 95 °C, followed by 34 cycles of 30 s at 95 °C, 30 s at 55–65 °C (depending on gene specific primers) and 60 s at 72 °C, then 10 min at 72 °C. The PCR products were purified from 1% agarose gel using a Universal DNA Purification kit (Tiangen, Beijing, China) and cloned into a pGEM-T Easy vector (Promega). Inserts were further sequenced for confirmation (BGI, Beijing, China).

For each GST gene, the complete open reading frame was cloned from SS and FR separately, and the sequenced results were subjected to alignment by DNAman software (DNAMAN 6.0, LynnonBioSoft, Quebec, QC, Canada). At least 10 samples from either SS or RS were sequenced for each gene.

### 3.5. Phylogenetic Analysis

Deduced amino acids of GSTs from *T. urticae*, *P. citri*, *I. scapularis*, and some other model insects were downloaded from the genome database of *T. urticae* and the NCBI database (http://www.ncbi.nlm.nih.gov/). The similarity of amino acids for each of the GSTs was determined using the DNAman program. The corresponding phylogenetic trees were determined by the neighbor-joining method, with 1000 bootstrap replications implemented in MEGA 4.0 [33].

### 3.6. Real-Time Quantitative PCR and Statistical Analysis

Primers for RT-qPCR study were designed by Primer Blast in NCBI. Primer sequences, amplicon sizes, PCR efficiencies, and coefficients of determination are presented in Table S1. Ribosomal protein S18 was used as a reference gene, as suggested by Sun in a stability evaluation study [34]. All qPCR reactions were performed on the Mx3000P thermal cycler (Stratagene, La Jolla, CA, USA). The 20 μL reaction system consisted of 1 μL of diluted cDNA, 10 μL GoTaq^®^ qPCR Master Mix (Promega), and 0.2 mM of each primer. Thermal cycling conditions consisted of an initial denaturation at 94 °C for 2 min, followed by 40 cycles of 94 °C for 15 s, and 60 °C for 30 s. After reaction, a melting curve analysis from 60 to 95 °C was applied to all reactions to ensure consistency and specificity of the amplified product.

The relative expression of GST genes was calculated using comparative *C*_t_ method based on the *C*_t_ value [35]. Data were statistically analyzed using a Student’s *t*-test and a one-way analysis of variance (ANOVA) test for significance (*p* = 0.05) in SPSS 12.0 (SPSS Inc., Chicago, IL, USA) for Windows.

## 4. Conclusions

Point mutation and overexpression were not involved in the fenpropathrin resistance mechanism of TcGSTs. Instead of maintaining a constantly high mRNA level in FR, three TcGSTs from the mu class turned out to be sensitive to fenpropathrin stimulation; Thus, FR mites could express detoxification enzymes in a timely manner to prevent damage. It is also believed to be a strategy to avoid extra energy costs involved in constant overexpression.

## Supplementary Materials

Supplementary table can be found at http://www.mdpi.com/1422-0067/15/12/22626/s1.
